# Airborne-Particle Abrasion vs. Hydrofluoric Acid Etching of Dental Ceramics: Impact on the Tensile Bond Strength

**DOI:** 10.3390/ma17235758

**Published:** 2024-11-25

**Authors:** Valerie Lankes, Andrea Coldea, John Meinen, Falk Schwendicke, Bogna Stawarczyk

**Affiliations:** 1 Department of Prosthetic Dentistry, University Hospital, LMU Munich, 80336 Munich, Germany; andrea.coldea@med.uni-muenchen.de (A.C.); john.meinen@med.uni-muenchen.de (J.M.); bogna.stawarczyk@med.uni-muenchen.de (B.S.); 2 Department of Conservative Dentistry and Parodontology, University Hospital, LMU Munich, 80336 Munich, Germany; falk.schwendicke@med.uni-muenchen.de

**Keywords:** feldspar, lithium silicate, zirconia, airborne particle abrasion, surface free energy, surface roughness, bond strength

## Abstract

This study evaluated whether airborne-particle abrasion could be an alternative to hydrofluoric acid etching as a pretreatment for the adhesive bonding of silicate ceramic restorations. Feldspar (FEL; n = 100), lithium silicate (LiSi; n = 100), and zirconia (ZrO_2_; (n = 80) substrates were CAD/CAM-fabricated and airborne-particle-abraded with Al_2_O_3_ (25 µm or 50 µm of mean particle size) at pressures of 0.05 or 0.1 MPa. The controls included FEL (60 s) and LiSi (20 s) etched with hydrofluoric acid. The surface free energy (SFE) and roughness (Ra) were measured. For the tensile bond strength (TBS), surfaces were conditioned using a primer (Monobond Plus) and luted to a resin composite (Variolink Esthetic). TBS was assessed initially (24 h, 37 °C water storage) and after thermocycling (5/55 °C, 10,000×). Statistical analysis (SPSS, V29) was performed using a one-way ANOVA, post hoc Scheffé, and a two-group *t*-test (*p* = 0.05). Abrasion with 50 µm and 0.1 MPa induced the highest Ra values across the materials (62.5 ± 3.88 µm). ZrO_2_ exhibited a higher TBS (35.4–49.5 MPa) than FEL and LiSi. For aged LiSi, the specimens treated at 0.1 MPa showed a higher TBS (18.7 ± 9.0 MPa) than those treated at 0.05 MPa, regardless of the particle size. The etched and aged FEL showed a higher SFE but a lower TBS compared to abrasion. Al_2_O_3_ particle abrasion (25 or 50 µm at 0.1 MPa) may replace etching for silicate-based ceramics, while 50 µm is recommended for ZrO_2_ at either pressure.

## 1. Introduction

Hydrofluoric acid (HF) is widely used in dentistry for etching silicate-based ceramics to enhance the bonding of restorations, such as crowns, bridges, and veneers [1]. However, the improper handling of HF can result in severe chemical burns to skin and eyes, posing significant risks to dental professionals and patients [2]. Additionally, HF can damage porcelain sinks and work surfaces, necessitating extreme caution when using this corrosive substance.

HF etches silicate-based ceramics by selectively dissolving the glassy (SiO_2_) matrix, creating a roughened surface that enhances micromechanical retention for bonding [3]. The etching pattern, optimal etching time (from 20 s to 240 s), and HF concentration (1–10%) depend on the ceramics’ composition [4,5,6]. Feldspar ceramics, such as leucite-reinforced or feldspathic ceramics, consist predominantly of a glassy matrix with dispersed crystalline phases like leucite. Lithium disilicate ceramics have a higher crystalline content, composed mainly of lithium disilicate crystals within a glassy matrix [7]. These compositional differences affect their mechanical, optical, and chemical properties, influencing the etching behavior and bonding protocols [8]. Despite the efficacy of HF etching, concerns about its toxicity and handling risks have led to the exploration of alternative surface treatments. Given that airborne-particle abrasion with alumina (Al_2_O_3_) particles is successfully used for surface cleaning and the roughening of most other dental materials [9], its application to silicate-based materials represents a logical alternative to explore.

Zirconia ceramics, due to their high crystallinity and lack of a glassy phase, are not susceptible to HF etching. Instead, airborne-particle abrasion with Al_2_O_3_ particles is employed to create micromechanical retention for bonding [10,11]. The effectiveness of airborne-particle abrasion depends on variables such as abrasive particle size, pressure, distance, angle of application, and duration. For delicate ceramic restorations, finer particles (e.g., 25–50 µm) are used to achieve adequate surface roughness without compromising the integrity of the restoration [12]. However, excessive airborne-particle abrasion pressure or inappropriate particle size may lead to surface damage or the alteration of the restoration’s fit [13]. Therefore, selecting appropriate airborne-particle abrasion factors is crucial for the effective and safe surface conditioning of zirconia restorations.

Besides micromechanical retention, chemical bonding is crucial for durable adhesion and for the stability and integrity of the restoration itself. Universal primers containing functional monomers like 10-methacryloyloxydecyl dihydrogen phosphate (MDP) facilitate chemical bonding to metal oxides and zirconia surfaces [14]. For silicate-based ceramics, silane coupling agents are used to enhance adhesion by forming covalent bonds between the silica in the ceramic and the resin matrix [15,16]. The combination of appropriate surface treatments and primers is essential to achieve optimal bond strength.

Earlier studies have reported acceptable bond strengths ranging from 7 to 15 MPa when silicate-based ceramics were airborne-abraded using alumina particles sized between 25 and 50 µm. However, these studies employed very high pressures (0.25–0.4 MPa), which could potentially damage the ceramic and compromise its mechanical properties [17,18,19,20].

Moreover, these studies often lacked artificial aging processes to simulate clinical conditions [21,22,23]. Simulating intraoral conditions is essential for evaluating the long-term durability of the ceramic–resin bond. Thermocycling is commonly used to mimic thermal stresses and water exposure in the oral environment by subjecting specimens to repeated temperature changes between 5 °C and 55 °C [24,25].

The aim of this study was to investigate whether silicate-based ceramics can be effectively bonded without the need for acid pretreatment, utilizing airborne-particle abrasion in conjunction with a ceramic primer. Additionally, the study aimed to evaluate the airborne-particle abrasion parameters for ZrO_2_ restorations. These tests were conducted in close proximity to clinical conditions, incorporating an artificial aging process. The null hypothesis posited that neither the alumina particle size nor the pressure would influence surface properties (surface free energy and surface roughness), and that neither the material composition, pretreatment variables (alumina particle size and pressure), nor aging would impact the tensile bond strength. Within silicate-based ceramics, it was assumed that there would be no difference in bond strength between etched and airborne-abraded surfaces. Comparing different airborne-particle abrasion parameters, such as the particle size and pressure, is clinically significant as these factors directly impact the mechanical properties. Moreover, selecting appropriate parameters for each type of restorative ceramic is essential to achieve sufficient bond strength while minimizing adverse effects on mechanical properties.

## 2. Materials and Methods

### 2.1. Specimen Preparation

**Figure 1:**
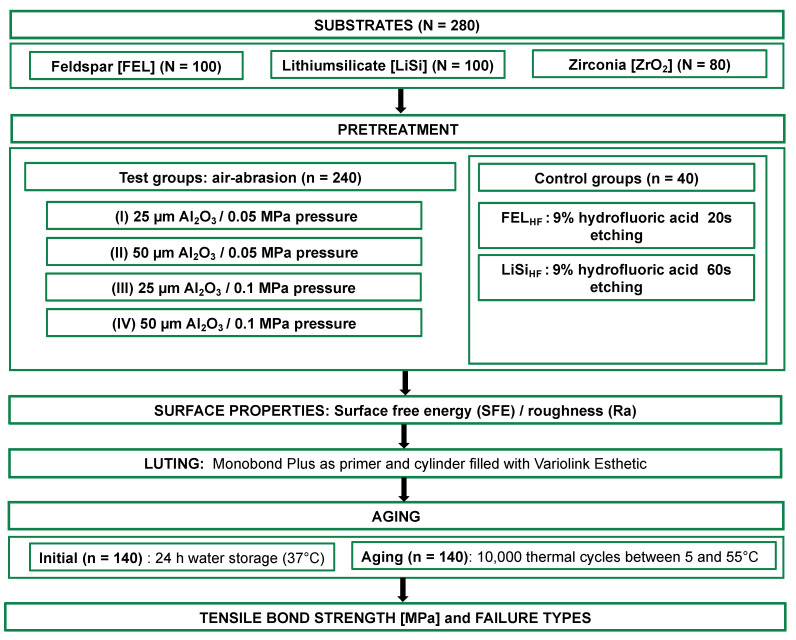
Overview of the study design.

In this in vitro study, three different CAD/CAM ceramics, (i) feldspar [FEL] (VITABLOCS Mark II, VITA Zahnfabrik, Bad Säckingen, Germany, Lot.Nr.: 93910), (ii) lithium silicate [LiSi] (IPS e.max CAD, Ivoclar, Schaan, Liechtenstein, Lot.Nr.: R49412), and (iii) 3 mol% yttria-stabilized tetragonal zirconia polycrystal (3Y-TZP) [ZrO_2_] (Lava Plus, Solventum, Seefeld, Germany Lot.Nr.: 468470), were investigated (Figure 1). Using an automatic precision cutting machine (Secotom-50, Struers, Ballerup, Denmark), the ceramic blocks were sectioned under water cooling into 280 equal-sized substrates (7.5 × 7.5 × 2 mm^3^; FEL: n = 100, LiSi: n = 100, ZrO_2_: n = 80). LiSi substrates, cut in their pre-crystallized state, were crystalli<ed according to the manufacturer’s instructions in a ceramic furnace (Astromat 624, Dekema Dental Ceramic Furnaces, Freilassing, Germany) at 840 °C with a 7-min holding time. ZrO_2_ substrates were air-dried at room temperature for 24 h post-cutting and then sintered at 1500 °C with a 30-min holding time, following manufacturer guidelines.

All substrates were embedded in acrylic resin (ScandiQuick A and B, ScanDia, Hagen, Germany; Lot Nos.: 616115 and 041225, respectively) and polished uniformly using silicon carbide (SiC) abrasive papers (P80 to P2000 grit) on an automatic polishing machine (Tegramin-20, Struers) under continuous water cooling. After polishing, the substrates of each ceramic were randomly assigned to four airborne-particle abrasion groups (n = 20 per group):

- Group I: 25 µm Al_2_O_3_ particles, 0.05 MPa pressure.
- Group II: 50 µm Al_2_O_3_ particles, 0.05 MPa pressure.
- Group III: 25 µm Al_2_O_3_ particles, 0.1 MPa pressure.
- Group IV: 50 µm Al_2_O_3_ particles, 0.1 MPa pressure.

Airborne-particle abrasion was performed using a Keramo 4 device (Renfert, Hilzingen, Germany) with a fixed 1.2 mm inner diameter nozzle. Al_2_O_3_ powder (Cobra, Renfert; Lot Nos.: 2546309 and 2548791 for 25 µm and 50 µm, respectively) was delivered at specified pressures. The nozzle was positioned 10 mm from the substrate surface at a 90° angle. Each specimen was manually moved in a circular motion under the nozzle for 10 s to ensure uniform airborne-particle abrasion.

The control groups FEL_HF_ and LiSi_HF_ (n = 20 each) were etched with 9% hydrofluoric acid gel (Ultradent Porcelain Etch, Ultradent, Brunnthal, Germany; Lot No.: BP6NG). The etching times were 60 s for FEL and 20 s for LiSi, following the established protocols. Post-etching, substrates were rinsed with distilled water for 30 s and ultrasonically cleaned in distilled water for 5 min.

### 2.2. Surface Free Energy (SFE) and Surface Roughness (Ra) Measurements

SFE was determined using the sessile drop method with a drop shape analysis system (Easy Drop DSA4, Krüss, Hamburg, Germany). Three drops each of distilled water and diiodomethane (Sigma-Aldrich, Bangalore, India, CAS No.: 75-11-6) were successively applied to each specimen at room temperature (23 °C). The contact angles were measured 5 s after drop application using the instrument’s software. The tangent-1 method was used for distilled water, and the circle-fitting method for diiodomethane. SFE was calculated using the Owens–Wendt–Rabel–Kaelble method, which considers both the polar and dispersive components of the surface energy.

Ra measurements were performed using a contact stylus profilometer (Perthometer M2, Mahr, Göttingen, Germany). A diamond stylus with a tip radius of 2 µm traced the surface profile over a length of 4 mm. For each specimen (n = 10 per group), six measurements were taken: three horizontally offset sections and three vertically offset sections.

All surfaces were inspected under a digital microscope (VHX-970F, Keyence, Osaka, Japan) at 200× magnification. FEL and LiSi groups were additionally examined using a scanning electron microscope (SEM) (Zeiss Supra 55 VP, Carl Zeiss, Oberkochen, Germany) at 1000× magnification.

### 2.3. Luting Procedure

Before luting, the substrates were cleaned ultrasonically for 3 min in 100% isopropanol (2-Propanol Ph.Eur., Otto Fischar, Saarbrücken, Germany) and then dried with compressed air. Monobond Plus (Ivoclar, Lot No.: Z04ZH9) was applied to the pretreated surfaces using a microbrush for 60 s and then air-dried. An acrylic cylinder (inner diameter: 2.9 mm) was placed on each pretreated surface and filled with a dual-curing resin composite cement (Variolink Esthetic, Shade: Neutral, Lot No.: Z04ZYG). Excess luting material was carefully removed with a microbrush before polymerization. The resin composite cement was polymerized for 40 s (10 s from each of four sides) using an LED light-curing unit (Elipar Deep Cure-S, Solventum) with a wavelength range of 430–480 nm and a light intensity of 1480 mW/cm^2^. The specimens were then stored in distilled water at 37 °C for 24 h. Additionally, half of the specimens underwent artificial aging through thermocycling using a thermocycler (SD Mechatronik, Feldkirchen-Westerham, Germany). The aging protocol consisted of 10,000 thermal cycles between 5 °C and 55 °C, with a dwell time of 20 s in each bath and a transfer time of 5 s between baths.

### 2.4. Tensile Bond Strength (TBS) Measurements

**Figure 2:**
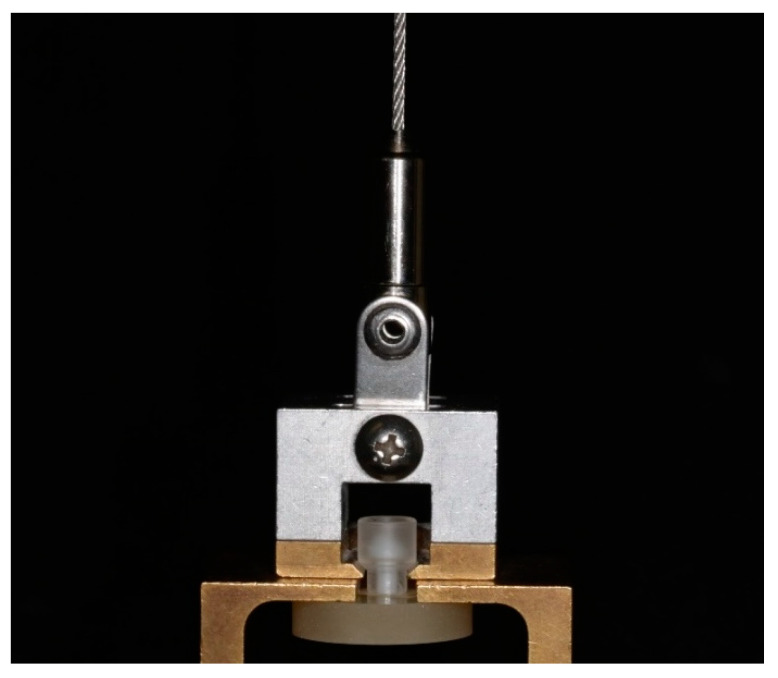
Specimen positioned in the TBS fixture.

Prior to TBS testing, the specimens were equilibrated at room temperature (23 °C) for one hour. TBS testing was conducted using a universal testing machine (Zwick 1445, Zwick, Ulm, Germany). Specimens were mounted in a custom-designed fixture that aligned the loading axis with the center of the bonded area to minimize shear forces and ensure pure tensile loading. The acrylic cylinder attached to the resin composite cement was connected to the upper grip of the testing machine, while the embedded substrate was fixed in the lower grip (Figure 2). A tensile load was applied at a crosshead speed of 5 mm/min until failure occurred. TBS was calculated by dividing the maximum load at failure by the cross-sectional area of the bonded interface (MPa).

The de-bonded surfaces were examined using a digital microscope (VHX-970F, Keyence, Osaka, Japan) at 75× magnification. Failure modes were categorized as:

- Adhesive failure (no resin composite cement remains on the substrate surface).
- Cohesive failure within the substrate.
- Cohesive failure within the resin composite cement.

### 2.5. Statistical Analysis

Data were analyzed using SPSS version 29.0 (IBM SPSS Statistics, Armonk, NY, USA). Descriptive statistics were calculated, and the Kolmogorov–Smirnov test assessed data normality. Statistical significance was set at *p* < 0.05. Data were analyzed using parametric statistical methods, as deviations from a normal distribution were less than 4%. One-way ANOVA with partial eta-squared (ηp^2^) was performed, followed by Scheffé post hoc tests for multiple comparisons. Pearson correlation analysis evaluated relationships between variables. Two-group *t*-tests assessed the impact of aging, pressure, and particle size. The frequency of failure types was analyzed using the chi-squared test.

## 3. Results

### 3.1. SFE, Ra, and SEM Analyses

The substrate had the greatest influence on SFE (*p* < 0.001, ηp^2^ = 0.587), followed by the pretreatment (*p* < 0.001, ηp^2^ = 0.358). Overall, ZrO_2_ exhibited lower SFE values compared to FEL and LiSi (*p* < 0.001). FEL_HF_ showed higher SFE values (*p* < 0.007) than the airborne-abraded substrates. For LiSi (*p* = 0.052) and ZrO_2_ (*p* = 0.121), the different pretreatment did not affect the SFE values (Table 1).

The substrate also had the greatest influence on Ra (*p* < 0.001, ηp^2^ = 0.988), followed by pretreatment (*p* < 0.001, ηp^2^ = 0.973), and the interaction between the substrate and pretreatment (*p* < 0.001, ηp^2^ = 0.899). The highest Ra values were observed with airborne-particle abrasion of 50 µm Al_2_O_3_ at 0.1 MPa (*p* < 0.001). For ZrO_2_ substrates, the pretreatment of 25 µm Al_2_O_3_ at 0.1 MPa resulted in higher Ra values than 50 µm at 0.05 MPa (*p* < 0.001). Airborne-particle abrasion with 25 µm Al_2_O_3_ particles at 0.05 MPa led to the lowest Ra values for FEL and ZrO_2_ groups (*p* < 0.001), whereas HF-etched substrates yielded the lowest Ra values for LiSi (*p* < 0.001). A positive correlation between SFE and Ra was detected (R = 0.284, *p* < 0.001).

**Figure 3:**
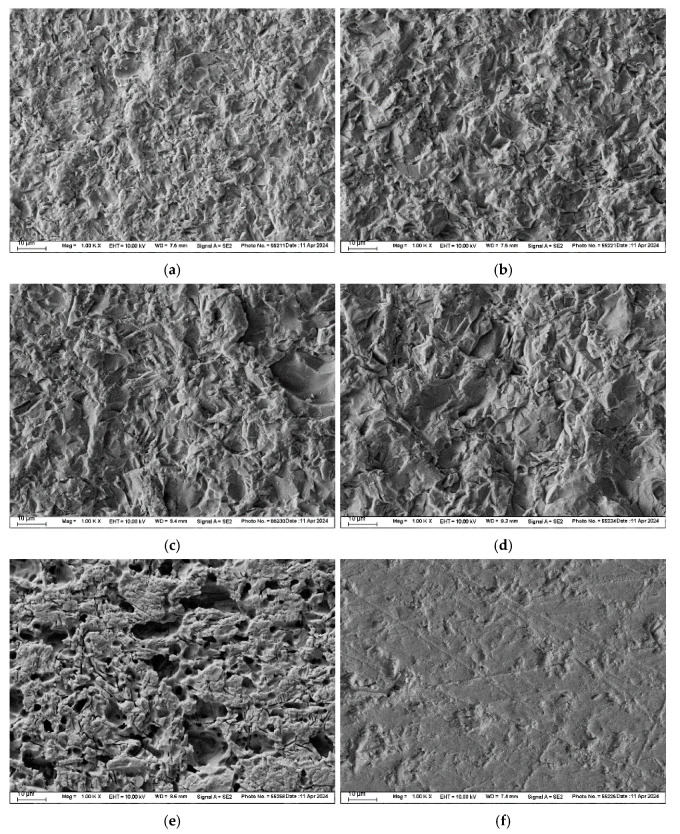
SEM images (magnification 1000×) of FEL substrates: (**a**) airborne-abraded with 25 µm Al_2_O_3_ at 0.05 MPa pressure, (**b**) airborne-abraded with 25 µm Al_2_O_3_ at 0.1 MPa pressure, (**c**) airborne-abraded with 50 µm Al_2_O_3_ at 0.05 MPa pressure, (**d**) airborne-abraded with 50 µm Al_2_O_3_ at 0.1 MPa pressure, (**e**) etched with 9% hydrofluoric acid, and (**f**) non-pretreated.

**Figure 4:**
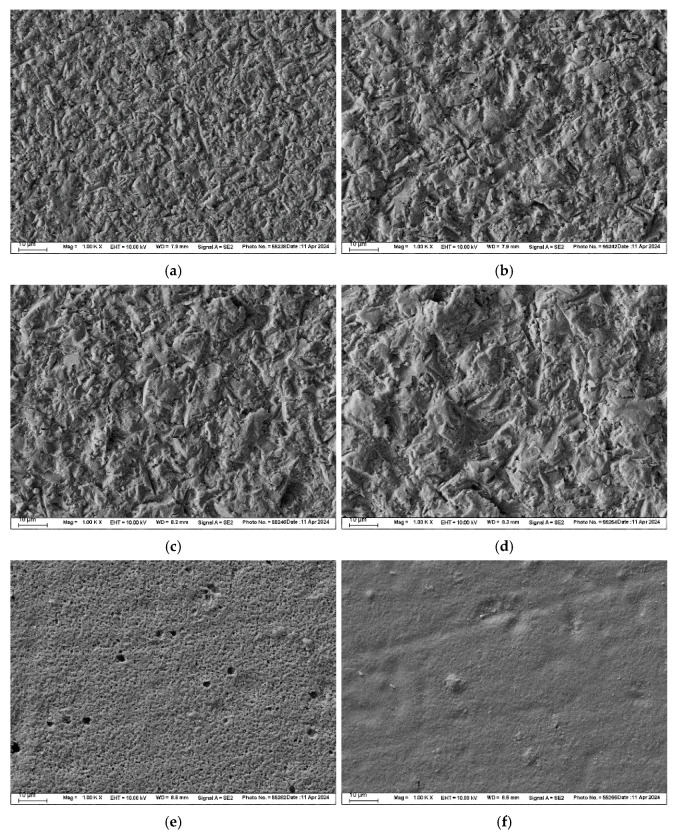
SEM images (magnification 1000×) of LiSi substrates: (**a**) airborne-abraded with 25 µm Al_2_O_3_ at 0.05 MPa pressure, (**b**) airborne-abraded with 25 µm Al_2_O_3_ at 0.1 MPa pressure, (**c**) airborne-abraded with 50 µm Al_2_O_3_ at 0.05 MPa pressure, (**d**) airborne-abraded with 50 µm Al_2_O_3_ at 0.1 MPa pressure, (**e**) etched with 9% hydrofluoric acid, and (**f**) non-pretreated.

The SEM images of FEL and LiSi illustrate abraded, etched, and non-treated surfaces (Figure 3 and Figure 4). Increasing abrasive particle size and pressure resulted in increased surface irregularities for both materials. Airborne-particle abrasion led to a more angular and fragmented surface topography compared to the etched surfaces, which displayed typical glassy-phase etching patterns.

### 3.2. TBS Measurements

The substrate had the greatest influence on TBS (*p* < 0.001, ηp^2^ = 0.755), followed by the interaction between the material and treatment (*p* < 0.001, ηp^2^ = 0.155), and the interaction between the aging level and pretreatment (*p* < 0.001, ηp^2^ = 0.139).

**Figure 5:**
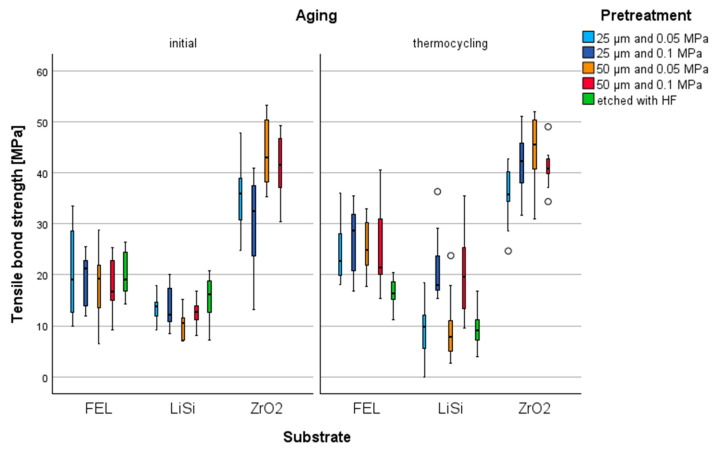
Boxplot of tensile bond strength values (MPa) of different Al_2_O_3_ airborne-abraded and etched (HF) substrates (FEL, LiSi, and ZrO_2_), initially and after thermocycling.

Among the substrates (Figure 5), ZrO_2_ exhibited the highest TBS values (*p* < 0.005). FEL substrates pretreated with 50 µm at 0.05 MPa, etched, and thermocycled FEL groups pretreated with 25 µm at 0.05 Mpa showed higher TBS values than LiSi substrates (*p* < 0.037).

Thermocycled FEL_HF_ substrates had lower TBS values (Table 2) compared to pretreatments with 25 µm/0.1 MPa and 50 µm/0.05 MPa (*p* < 0.039). For the initially tested LiSi substrates, pretreatment with HF acid resulted in the highest TBS values, whereas pretreatment with 50 µm/0.05 MPa led to the lowest values (*p* < 0.030). The aged LiSi substrates treated with 0.1 MPa showed higher TBS values (*p* < 0.016) compared to the etched and 0.05 MPa pretreated LiSi groups. In the initial state, FEL substrates treated with 50 µm/0.05 MPa and 50 µm/0.1 MPa, as well as the aged substrates treated with 50 µm/0.05 MPa, exhibited higher TBS values than ZrO_2_ (*p* < 0.018). In contrast, initial 25 µm/0.1 MPa and the aged 25 µm/0.05 MPa ZrO_2_ substrates showed lower TBS values (*p* < 0.009).

Regarding the aging regime, pretreatment with 25 µm/0.1 MPa resulted in higher TBS values after thermocycling (*p* < 0.004). Pretreatment with 50 µm/0.1 MPa showed higher TBS values for FEL and LiSi substrates after thermocycling (*p* < 0.017), and for FEL, the aged substrates treated with 50 µm/0.05 MPa exhibited higher values (*p* = 0.008). Pretreatment with HF acid led to initially higher TBS values (*p* < 0.023); additionally, for LiSi substrates, pretreatment with 25 µm/0.05 MPa showed higher values (*p* = 0.023).

Concerning the Al2O3 particle size (Table 2), the initial LiSi substrates treated with 0.05 MPa and 25 µm showed increased TBS values (*p* = 0.011). Al_2_O_3_ particles of size 50 µm increased TBS values for ZrO_2_ substrates treated with 0.05 MPa (*p* < 0.005) and, initially, for those treated with 0.1 MPa (*p* = 0.002).

In terms of the pressure level, for the aged LiSi substrates, a pressure level of 0.1 MPa increased TBS values (*p* < 0.003). The aged ZrO_2_ substrates treated with Al_2_O_3_ 25 µm showed higher TBS values with a pressure level of 0.1 MPa (*p* = 0.013).

### 3.3. Failure Types

**Figure 6:**
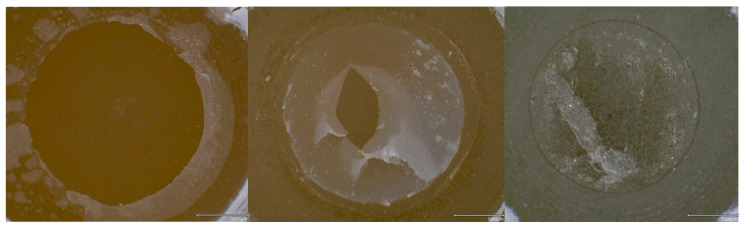
Overview of failure types: adhesive failure between LiSi substrate (**left**), cohesive failure within the resin composite cement on a LiSi substrate (**middle**), and cohesive failure within a FEL substrate (**right**).

FEL specimens predominantly exhibited cohesive failures within the substrate (20–100%, Table 3 and Figure 6). Thermocycled LiSi substrates showed cohesive failures within the resin composite cement (40–80%). ZrO_2_ substrates exclusively displayed cohesive failures within the resin composite cement (100%).

## 4. Discussion

In this study, the impact of various airborne-particle abrasion parameters compared to etching using HF acid on the surface properties and tensile bond strength of CAD/CAM ceramics (feldspar (FEL), lithium silicate (LiSi), and zirconia (ZrO_2_)) was investigated. The first and second null hypotheses were rejected based on the results obtained.

ZrO_2_ exhibited lower surface free energy (SFE) values compared to FEL and LiSi, which could be attributed to the inherent material characteristics. The microstructure of ZrO_2_ may undergo fewer changes, resulting in smaller increases in the surface area and subsequently lower SFE values. For ZrO_2_, airborne-particle abrasion could lead to a surface phase transformation from the tetragonal to monoclinic phases, enhancing toughness through transformation toughening without necessarily increasing SFE [26]. Smaller particles have a limited ability to remove material from the surface due to their lower mass and momentum upon impact. High-pressure abrasion with larger particles can induce microcrack formation and surface damage due to the high-energy impact, inadvertently increasing surface roughness [27]. The angle at which the alumina particles hit the silicate-based surface could also play a role.

HF acid preferentially attacks the glassy matrix of ceramics. In FEL, the etching process removes the glassy phase more aggressively, exposing and undercutting the crystalline phases, as evidenced in the SEM images compared to LiSi. This results in a rougher surface with initially higher tensile bond strengths, as the etching process leaves behind protruding crystalline components and deeper pits [28]. The structure of LiSi allows for controlled removal of both the glass matrix around the crystalline phases, resulting in a more uniform etch pattern with less pronounced topographical variations compared to mechanical abrasion [4]. Airborne-particle abrasion tends to create a roughened surface through pitting and micro-fracturing, it may not achieve the same depth or irregularity of features as chemical etching, leading to comparatively lower roughness values. The standard roughening procedure for FEL and LiSi restorations is hydrofluoric acid etching. Zirconia is usually roughened by a 50 µm alumina particle size with a maximum of 0.25 MPa pressure [29]. Therefore, in this study, the particle size was chosen based on the parameters used for zirconia and compared to a lower particle size of 25 µm as well as reduced pressures of 0.1 and 0.05 MPa.

The Pearson correlation suggests a chemical bond, as the tensile bond strength increases despite low SFE and Ra values (*p* < 0.001). Monobond Plus is a universal primer designed to enhance bonding between dental restorations materials and resin composite cements. It contains components such as silane coupling agents and phosphoric acid monomers. Silane molecules form covalent bonds with silica on ceramic surfaces [30], while phosphoric acid monomers bond to the oxide layer on ZrO_2_ surfaces, forming phosphate esters that improve adhesion between ZrO_2_ and resin composite cement [31]. Apart from the bond, the chemical adhesion is crucial for the overall stability, as it enhances the structural integrity of the connection and ensures the long-term stability of the restoration. A strong chemical bond prevents micro-movements at the interface, which reduces wear and minimizes the risk of fractures or failures of the crown [32].

Thermocycling simulates oral environment thermal stresses over time, introducing water into the ceramic–resin composite cement interface. Water can degrade silane coupling agents and the resin matrix, weakening bonds. Rapid temperature changes induce thermal stress within the bonded restorations [33], potentially causing microcracks in the adhesive layers or resin composite cement. Previous studies in the dental field applied 5000 to 10,000 thermal cycles. According to a review article [34], 10,000 cycles are estimated to correspond to approximately one year in vivo. For LiSi substrates, airborne-particle abrasion can create deeper micro-retentions than etching, providing mechanical anchorage less susceptible to hydrolytic degradation under thermocycling conditions, resulting in increased bond strength. While hydrofluoric acid etching creates microporous surfaces for good initial adhesion [35], these surfaces may be more prone to degradation from water infiltration and thermal stress over time. Increased bond strength can be explained by the higher temperature, which can promote post-polymerization of the luting area [36].

A notable finding was the predominance of cohesive fractures in FEL substrates when airborne-abraded, particularly at 0.1 MPa pressure, compared to HF etching. Cohesive fractures suggest that the failure occurred within the ceramic material itself rather than at the interface between the ceramic and the resin composite cement. This pattern indicates that airborne-particle abrasion, while enhancing bond strength, might introduce microcracks that compromise the integrity of the substrate. This is especially significant for FEL, which inherently has a lower flexural strength compared to LiSi and ZrO_2_. These results are consistent with earlier studies, which found that a higher abrasive pressure (<0.28 MPa) resulted in a lower flexural strength of a FEL ceramic compared to a treatment with hydrofluoric acid [17]. However, studies have also shown that the duration of hydrofluoric acid treatment and the concentration also affect the flexural strength [8]. In contrast, LiSi and ZrO_2_ substrates exhibited fewer or no cohesive fractures, likely due to their higher flexural strength. The durability of LiSi under airborne-particle abrasion was evident, as cohesive failures were minimal, and when present, they were often confined to the resin composite cement rather than the ceramic itself. For ZrO_2_, which is known for its superior mechanical properties, cohesive failures were virtually absent, underscoring its resilience even under an abrasive pretreatment. The relationship between cohesive and adhesive failures provides valuable insights into the durability and clinical reliability of airborne-particle abrasion. Cohesive failures within the substrate, while indicative of strong bonding, raise concerns about the potential for substrate weakening, especially for materials like FEL. On the other hand, adhesive failures, which occur at the interface, may suggest suboptimal bond integrity.

Airborne alumina particle abrasion offers clinical benefits for aesthetic restorative materials. It enhances bond strength and increases surface roughness, both of which are crucial for the improved retention and stability of dental restorations. Nevertheless, achieving optimal marginal adaptation remains pivotal for clinical success and is closely related to the specific type of restorative material used [37].

To make definitive clinical recommendations, future studies should include flexural strength measurements of substrates following various surface treatments to assess potential structural weakening from airborne-particle abrasion procedures. Understanding the long-term outcomes associated with these treatments is crucial for ensuring their reliability in clinical practice. Airborne-particle abrasion, while offering a safer alternative to hydrofluoric acid (HF) etching, may introduce microcracks that weaken the substrate, particularly in materials with a lower inherent flexural strength. This micro-damage could affect the longevity and stability of restorations under functional loads. Additionally, residual stresses may develop due to milling and airborne-particle abrasion, leading to surface plasticity and compressive stresses. These stresses, however, can be mitigated or eliminated through etching, contributing to the improved durability of the restorations.

The present study shows that silicate-based ceramics can be airborne-particle-abraded to achieve similar or even higher bond strengths than after conventional etching with hydrofluoric acid. There are many benefits to clinical practice if the pretreatment of silicate-based ceramics were similar to that variety of dental materials, including alloys, polyether ether ketone (PEEK), polymethylmethacrylate (PMMA), and composites, which require airborne-particle abrasion prior to adhesive cementation [38,39,40]. Airborne-particle abrasion can be performed quickly, allowing for a more uniform approach to preparing different materials and does not require different etching times for various compositions of silicate-based ceramics. Eliminating hydrofluoric acid through airborne-particle abrasion would reduce the risk of chemical burns, inhalation of toxic fumes, and other safety risks associated with acid etching amongst practitioners and for patients during the intraoral repair of silicate-based restorations. This change would increase safety in the dental office and reduce the need for strict safety precautions and specialized disposal protocols.

## 5. Conclusions

Due to its highly corrosive and toxic properties, the improper handling of hydrofluoric acid poses significant risks for dentists and patients. The airborne-particle abrasion of silicate-based ceramic restorations—such as feldspar ceramic (FEL) and lithium disilicate (LiSi)—with Al_2_O_3_ particles of mean sizes of 25 and 50 µm at a pressure of 0.1 MPa can serve as an effective pretreatment method for adhesive bonding, potentially replacing hazardous acid etching. However, further research is needed to evaluate the extent of surface damage and the resulting decrease in the mechanical properties of silicate-based ceramics due to airborne-particle abrasion, even at lower pressures of 0.05 MPa. Zirconia (ZrO_2_) is typically airborne-abraded as a standard pretreatment method before adhesive bonding and should be treated using 50 µm alumina powder. The pressure applied during airborne-particle abrasion does not appear to have a significant impact on bond strength values for zirconia.

## Figures and Tables

**Table 1:** Descriptive statistics (mean ± standard deviation (SD)) and 95% confidence intervals (CI) of the measured surface energy SFE and roughness Ra [µm] on different Al_2_O_3_ airborne-abraded and etched (HF) substrates.

		SFE		Ra	
Substrate	Pretreatment	Mean ± SD	95 CI %	Mean ± SD	95 CI %
FEL	25 µm 0.05 MPa	63.1 ± 1.99 ^z^	(61.5; 64.5)	0.44 ± 0.03 ^z^	(0.43; 0.47)
	50 µm 0.05 MPa	57.6 ± 1.63 ^z^	(56.3; 58.8)	0.73 ± 0.03 ^x^	(0.70; 0.75)
	25 µm 0.1 MPa	57.8 ± 7.52 ^z^	(52.3; 63.3)	0.71 ± 0.03 ^x^	(0.68; 0.74)
	50 µm 0.1 MPa	60.5 ± 3.97 ^z^	(57.5; 63.4)	1.18 ± 0.05 ^w^	(1.13; 1.22)
	FEL_HF_	70.7 ± 3.11 ^y^	(68.3; 72.9)	0.68 ± 0.06 ^y^	(0.59; 0.69)
LiSi	25 µm 0.05 MPa	64.9 ± 4.12 ^z^	(61.8; 67.9)	0.18 ± 0.03 ^x^	(0.14; 0.21)
	50 µm 0.05 MPa	59.2 ± 3.92 ^z^	(56.2; 62.0)	0.34 ± 0.03 ^y^	(0.30; 0.36)
	25 µm 0.1 MPa	58.8 ± 8.83 ^z^	(52.4; 65.2)	0.33 ± 0.02 ^y^	(0.31; 0.36)
	50 µm 0.1 MPa	62.5 ± 3.88 ^z^	(59.5; 65.3)	0.61 ± 0.03 ^z^	(0.58; 0.64)
	LiSi_HF_	62.2 ± 6.90 ^z^	(62.1; 72.3)	0.07 ± 0.01 ^z^	(0.05; 0.07)
ZrO_2_	25 µm 0.05 MPa	49.5 ± 4.50 ^z^	(46.1; 52.7)	0.10 ± 0.01 ^w^	(0.08; 0.11)
	50 µm 0.05 MPa	45.2 ± 5.12 ^z^	(41.4; 48.9)	0.13 ± 0.02 ^x^	(0.10; 0.15)
	25 µm 0.1 MPa	48.8 ± 1.74 ^z^	(47.4; 50.0)	0.18 ± 0.06 ^y^	(0.15; 0.17)
	50 µm 0.1 MPa	49.2 ± 2.92 ^z^	(47.0; 51.4)	0.27 ± 0.01 ^z^	(0.25; 0.28)

^zyxw^: different letters represent significant differences between one material and pretreatment groups for SFE and Ra.

**Table 2:** Descriptive statistics (mean ± standard deviation (SD)) and 95% confidence intervals (CI) of TBS [MPa] initial and after aging on different Al_2_O_3_ airborne-abraded and etched (HF) substrates.

		TBS			
Substrate	Pretreatment	Initial		After Artificial Aging	
		Mean ± SD	95 CI %	Mean ± SD	95 CI %
FEL	25 µm 0.05 MPa	20.1 ± 8.1 ^aAIiα^	(13; 26)	24.8 ± 6.3 ^bABIiα^	(19; 30)
	50 µm 0.05 MPa	18.3 ± 6.5 ^bAIiα^	(12; 23)	25.3 ± 5.2 ^bBIIiα^	(20; 29)
	25 µm 0.1 MPa	18.9 ± 5.0 *^aAIiα^	(14; 23)	26.8 ± 6.8 ^aBIIiα^	(20; 32)
	50 µm 0.1 MPa	17.8 ± 5.2 ^aAIiα^	(13; 22)	24.9 ± 8.2 ^aABIIiα^	(17; 31)
	FEL_HF_	19.8 ± 4.5 ^bAII^	(16; 23)	16.2 ± 3.0 ^bAI^	(13; 19)
LiSi	25 µm 0.05 MPa	13.3 ± 2.7 ^aABIIiiα^	(10; 16)	9.3 ± 5.2 ^aAIiα^	(4; 14)
	50 µm 0.05 MPa	10.2 ± 2.8 ^aAIiα^	(7; 13)	9.7 ± 6.6 ^aAIiα^	(3; 15)
	25 µm 0.1 MPa	12.7 ± 3.4 ^aABIiα^	(9; 16)	21.1 ± 6.8 *^aBIIiβ^	(15; 26)
	50 µm 0.1 MPa	12.5 ± 2.7 ^aABIiα^	(9; 15)	18.7 ± 9.0 ^aBIIiβ^	(11; 26)
	LiSi_HF_	15.4 ± 4.2 ^aBII^	(12; 19)	9.7 ± 4.1 ^aAI^	(5; 13)
ZrO_2_	25 µm 0.05 MPa	35.4 ± 6.8 ^bABIiα^	(29; 41)	35.7 ± 5.6 ^cAIiα^	(30; 43)
	50 µm 0.05 MPa	43.7 ± 6.4 ^cBIiiα^	(38; 49)	44.4 ± 6.8 ^bBIiiα^	(38; 50)
	25 µm 0.1 MPa	29.5 ± 9.3 ^bAIiα^	(21; 37)	42.1 ± 6.1 ^bABIIiβ^	(36; 47)
	50 µm 0.1 MPa	41.4 ± 6.1 ^bBIiiα^	(36; 46)	41.0 ± 3.9 ^bABIiα^	(37; 44)

* not normally distributed. ^abc^: different lowercase letters present significant differences between the substrates within one pretreatment and aging group; ^AB^: different letters present significant differences between pretreatments within one substrate and aging group; ^I,II^: different letters present significant differences between the aging regime within one substrate and pretreatment group; ^i,ii^: different letters present significant differences between the applied powder particle size within one pressure, substrate, and aging group; ^αβ^: different uppercase letters present significant differences between the applied pressure within one particle size, substrate, and aging group.

**Table 3:** Descriptive statistics for failure types of all tested groups.

Substrate	Pretreatment	% Adhesive and 95% CI	% Cohesive Within the Substrate and 95% CI	% Cohesive Within the Resin Composite and 95% CI
		**Initial**		
FEL	Al_2_O_3_ 25 µm 0.05 MPa	0 (0;31) ^a^	70 (35;93) ^b^	30 (7;65) ^a^
	Al_2_O_3_ 50 µm 0.05 Mpa	0 (0;31) ^a^	60 (26;88) ^b^	40 (12;74) ^b^
	Al_2_O_3_ 25 µm 0.1 Mpa	0 (0;31) ^a^	100 (69;100) ^b^	0 (0;31) ^a^
	Al_2_O_3_ 50 µm 0.1 Mpa	0 (0;31) ^a^	60 (26;88) ^b^	40 (12;74) ^b^
	FEL_HF_	0 (0;31) ^a^	50 (19;82) ^b^	50 (19;82) ^b^
LiSi	Al_2_O_3_ 25 µm 0.05 Mpa	90 (56;100) ^b^	10 (0;45) ^a^	0 (0;31) ^a^
	Al_2_O_3_ 50 µm 0.05 Mpa	90 (56;100) ^b^	0 (0;31) ^a^	10 (0;45) ^a^
	Al_2_O_3_ 25 µm 0.1 Mpa	40 (12;74) ^b^	0 (0;31) ^a^	60 (26;88) ^b^
	Al_2_O_3_ 50 µm 0.1 Mpa	20 (3;56) ^a^	0 (0;31) ^a^	80 (44;97) ^b^
	LiSi_HF_	0 (0;31) ^a^	0 (0;31) ^a^	100 (69;100) ^b^
ZrO_2_	Al_2_O_3_ 25 µm 0.05 Mpa	0 (0;31) ^a^	0 (0;31) ^a^	100 (69;100) ^a^
	Al_2_O_3_ 50 µm 0.05 Mpa	0 (0;31) ^a^	0 (0;31) ^a^	100 (69;100) ^a^
	Al_2_O_3_ 25 µm 0.1 Mpa	0 (0;31) ^a^	0 (0;31) ^a^	100 (69;100) ^a^
	Al_2_O_3_ 50 µm 0.1 Mpa	0 (0;31) ^a^	0 (0;31) ^a^	100 (69;100) ^a^
		**After aging**		
FEL	Al_2_O_3_ 25 µm 0.05 Mpa	0 (0;31) ^a^	20 (3;56) ^a^	80 (44;97) ^b^
	Al_2_O_3_ 50 µm 0.05 Mpa	0 (0;31) ^a^	50 (19;82) ^b^	50 (19;82) ^b^
	Al_2_O_3_ 25 µm 0.1 Mpa	0 (0;31) ^a^	50 (19;82) ^b^	50 (19;82) ^b^
	Al_2_O_3_ 50 µm 0.1 Mpa	0 (0;31) ^a^	70 (35;93) ^b^	30 (7;65) ^a^
	FEL_HF_	0 (0;31) ^a^	50 (19;82) ^b^	50 (19;82) ^b^
LiSi	Al_2_O_3_ 25 µm 0.05 Mpa	40 (12;74) ^a^	20 (3;56) ^a^	40 (12;74) ^a^
	Al_2_O_3_ 50 µm 0.05 Mpa	20 (3;56) ^a^	0 (0;31) ^a^	80 (44;97) ^b^
	Al_2_O_3_ 25 µm 0.1 Mpa	10 (0;45) ^a^	10 (0;45) ^a^	80 (44;97) ^b^
	Al_2_O_3_ 50 µm 0.1 Mpa	20 (3;56) ^a^	0 (0;31) ^a^	80 (44;97) ^b^
	LiSi_HF_	10 (0;45) ^a^	10 (0;45) ^a^	80 (44;97) ^b^
ZrO_2_	Al_2_O_3_ 25 µm 0.05 Mpa	0 (0;31) ^a^	0 (0;31) ^a^	100 (69;100) ^b^
	Al_2_O_3_ 50 µm 0.05 Mpa	0 (0;31) ^a^	0 (0;31) ^a^	100 (69;100) ^b^
	Al_2_O_3_ 25 µm 0.1 Mpa	0 (0;31) ^a^	0 (0;31) ^a^	100 (69;100) ^b^
	Al_2_O_3_ 50 µm 0.1 Mpa	0 (0;31) ^a^	0 (0;31) ^a^	100 (69;100) ^b^

^ab^ Different lowercase letters present differences in the 95% CI within one substrate, pretreatment, and aging group.

## Data Availability

The original contributions presented in this study are included in the article. Further inquiries can be directed to the corresponding author.
